# *Eurycoma longifolia*: Medicinal Plant in the Prevention and Treatment of Male Osteoporosis due to Androgen Deficiency

**DOI:** 10.1155/2012/125761

**Published:** 2012-07-15

**Authors:** Nadia Mohd Effendy, Norazlina Mohamed, Norliza Muhammad, Isa Naina Mohamad, Ahmad Nazrun Shuid

**Affiliations:** Department of Pharmacology, Faculty of Medicine, The National University of Malaysia, Kuala Lumpur Campus, 50300 Kuala Lumpur, Malaysia

## Abstract

Osteoporosis in elderly men is now becoming an alarming health issue due to its relation with a higher mortality rate compared to osteoporosis in women. Androgen deficiency (hypogonadism) is one of the major factors of male osteoporosis and it can be treated with testosterone replacement therapy (TRT). However, one medicinal plant, *Eurycoma longifolia* Jack (*EL*), can be used as an alternative treatment to prevent and treat male osteoporosis without causing the side effects associated with TRT. *EL* exerts proandrogenic effects that enhance testosterone level, as well as stimulate osteoblast proliferation and osteoclast apoptosis. This will maintain bone remodelling activity and reduce bone loss. Phytochemical components of *EL* may also prevent osteoporosis via its antioxidative property. Hence, *EL* has the potential as a complementary treatment for male osteoporosis.

## 1. Introduction

Traditional medicine is defined by the World Health Organization as the sum total of knowledge, skills, and practices based on the theories, beliefs, and experience indigenous to different cultures that are used to maintain health, as well as to prevent, diagnose, improve, or treat physical and mental illnesses [1]. It covers numerous alternatives that vary from country to country and is referred to as “alternative” or “complementary” medicine. Traditional medicine comprises of biologically based therapies (e.g., herbs and vitamins supplement), manipulative and body-based therapies (e.g., massage and reflexology), mind-body medicine (e.g., meditation), and holistic medical systems, such as acupuncture and ayurveda [2].

For centuries, medicinal plants (i.e., herbal medicine) have been a part of cultural heritage. More than 35,000 plant species have been reported to be used in various human cultures around the world for medicinal purposes [3]. Burkill, in his extensive compilation of economic products of the Malay peninsula, recorded not less than 1,300 plants, which were used in traditional medicine [4]. In spite of the great advances observed in modern medicine in recent decades, plants still make an important contribution to health care. Nowadays, medicinal plants play a significant role as an alternative medicine due to the damaging effects of food processing, environment, and hazardous side effects of prolonged medications [5, 6]. 

The earliest report on medicinal plant research in Malaysia was on the phytochemical screening of 205 plants in Sabah [7, 8], followed a few years later by the screening of 200 plant species in Peninsular Malaysia for the presence of alkaloids [9]. Subsequently, more plants were screened chemically for alkaloids, saponins, triterpenes, and steroids [10] to the extent of becoming the focus of major worldwide attention currently. Malaysia is rated as one of the 12 countries in the world with mega diversity of plants [11, 12].

**Figure 1:**
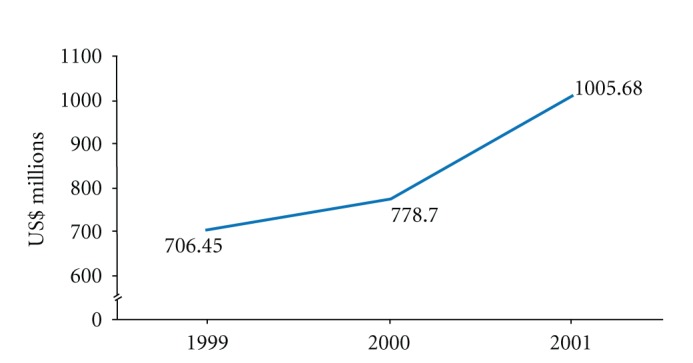
Sales of herbal medicine of nine representative countries. It is shown that between 1999 and 2001 alone, the sales value of herbal medicines in this group of countries increased by more than 40%. (Source: [13]). Growth in the sales of herbal medicines in a group of nine representative countries, 1999–2001 (Bhutan, Canada, the Czech Republic, Iran, Madagascar, Malaysia, Pakistan, Sudan, and Sweden).

According to a WHO global survey report, Malaysia was one of the nine countries that contributed a large amount of sales in herbal medicine worldwide between the year of 1999 to 2001, as seen in Figure 1 [13]. In 2008, the Malaysian market for herbal and natural products was estimated to worth approximately RM10 billion, increasing at the rate of 8% per year. Hence, it was projected that by 2050, the global market for herbal products would be around 5 US trillion dollars [14]. Examples of famous Malaysian medicinal plants are *Eurycoma longifolia *Jack (Tongkat Ali), *Labisia pumila* (Kacip Fatimah), *Piper sarmentosum* (Daun kaduk), *Ficus deltoidea* (Mas cotek), and *Centella Asiatica *(Pegaga). These herbs are known to exert antibacterial, antipyretic, antioxidant, anti-inflammatory, and potential antitumor activities [15–17].

## 2. *Eurycoma longifolia* Jack

*Eurycoma longifolia *Jack (*EL*) or commercially known as Tongkat Ali in Malaysia, Pasak bumi in Indonesian, Piak and Tung saw in Thailand, and Cay ba binh in Vietnam [18] is a famous medicinal plant in the family of Simaroubaceae. Besides *EL*, there are three other plant species also known locally as Tongkat Ali which literally means “Stick of a man” and “Ali” of which referring to its aphrodisiac property. The three plant species are *Entomophthora apiculata*, *Polyathia bullata*, and *Goniothalamus *sp. [19, 20]. However, *EL* is the most widely used species for its therapeutic activities. In Malaysia, *EL* is well known among various ethnic groups for treating disease and enhancing health, particularly sexual health among men. *EL* is a shrub tree that grows up to 10 metres in height, with long leaves that are green in colour. The leaves are pinnate in shape (i.e., the leaflets are arranged in pairs) [21]. The flowers of this tree are dioecious, whereas its ovoid-shaped fruits will turn to dark brown colour when they are ripe [22]. Due to the high demand of *EL* for its tremendous health benefits, *EL* preparations are now widely available in the health-food market in the form of raw crude powder where the root is dried and grinded without involving any other chemical processing steps.*EL* is also available in the form of capsules which may either contain raw crude powder or standardised *EL* extract. *EL* extract is prepared by extracting the active ingredients, adjusting the preparation to a defined content of a constituent, and followed by concentrating it to a standard level. Other than that, *EL* is available as an additive brewed with coffee and even canned processed drinks [23, 24]. It has been recommended that *EL* should be administered orally, as other means such as intraperitoneal could enhance its toxicity by approximately 100-fold [25].

**Figure 2:**
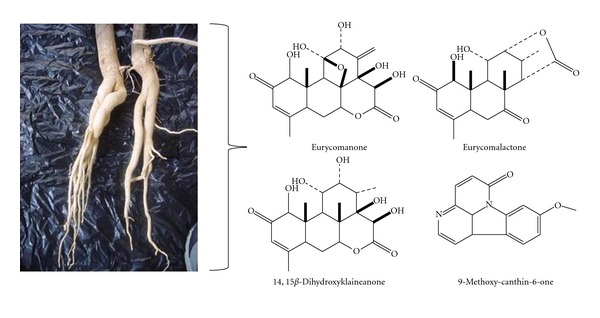
Some of the chemical constituents isolated from the root of *Eurycoma longifolia *Jack (Source: [27]).

A wide range of chemical compounds have been isolated, especially from the root of *EL*, which include eurycomanone, eurycomanol, eurycomalactone, canthine-6-one alkaloid, 9-hydroxycanthin-6-one, 14,15*β*-dihydroxyklaineanone, phenolic components, tannins, quanissoids, and triterpenes, as depicted in Figure 2 [26, 27]. Due to the presence of these chemical compounds, the root has been reported to have effective medicinal values in terms of sexual enhancement property for males, as well as antipyretic, antimalarial, antibacterial, and antitumor properties [28, 29]. *EL* has been well documented to exert antioxidative properties due to its high concentrations of superoxide dismutase (SOD) [30, 31]. *EL* is famously known for its aphrodisiac effect, which is due to its ability to stimulate the production or action of androgen hormones, especially testosterone. Hence, it can be used as an alternative for testosterone replacement therapy [21] in a variety of related conditions, for example, in the treatment of male osteoporosis due to androgen deficiency [32].

To the best of our knowledge, till date, there is no reliable review on the effects of *Eurycoma longifolia *Jack on bone remodeling and its antiosteoporotic value. Hence, this review focused on the effects of *Eurycoma longifolia *Jack on bone and its postulated antiosteoporotic mechanisms in treating osteoporosis due to androgen deficiency. This information will be useful and applicable for future researches on osteoporosis and the development of a more comprehensive natural medicine approach to bone diseases.

## 3. Osteoporosis

The incidences of osteoporosis and osteoporosis-related fractures are increasing and have now become a major public health issue. According to the World Health Organization (WHO), osteoporosis occurs if the bone mineral density is more than 2.5 standard deviation below the peak bone mass reference standard for young women. If a similar criterion is used for men in United States (by referring to the peak bone mass reference standard for young men, instead for young women), approximately 1-2 million men may have osteoporosis and another 8–13 million may have osteopenia [33]. 

Osteoporosis is categorized as one of the serious chronic diseases that has become a significant socioeconomic burden in many countries. Chronic diseases are a major factor in the continuous growth of medical care spending [34]. According to the WHO, chronic diseases are by far the leading cause of mortality in the world [35]. Osteoporosis is a silent, slowly progressive systemic skeletal disease that is characterized by low bone mass and microarchitectural deterioration of bone tissue. It does not usually present with a significant symptom until a bone fracture occurs, hence, the so-called “silent” disease [36]. Therefore, an acute condition due to bone fracture may occur, although osteoporosis is classified as a chronic disease due to its slow and destructive progression. There are two types of this disease: primary and secondary osteoporosis. Primary osteoporosis can be caused by aging and hormonal disturbance. It is also known as senile osteoporosis or postmenopausal osteoporosis, which mainly affects women [37]. On the other hand, secondary osteoporosis is caused by exogenous factors. These include diseases affecting bone metabolism, such as Cushing's disease, hyperparathyroidism, and liver disease. Medications, such as steroid (prednisone), barbiturates, thyroxine, heparin, and diabetic medications, may also impair bone metabolism, leading to bone loss [38].

Bone is continuously remodelled throughout life, whereby bone resorption activity by osteoclasts is always followed by bone formation by osteoblasts in a perpetual cycle [39]. Osteoporosis occurs when the bone formation and bone resorption cycle is impaired. The resorption and reversal phases of bone remodelling are short, while the period required for osteoblastic replacement of the bone is long. Therefore, any factors that increase the rate of bone remodelling, such as hormonal deficiency, will result in loss of bone mass [40].

The sex hormones, androgen and estrogen, play an important role in regulating bone health. Androgens are the most abundant circulating sex steroids in both men and women that modulate bone remodelling cycle through direct androgenic activity via androgen receptor (AR) or indirect action through aromatization to estrogens [41]. Testosterone is the main androgen and predominant sex steroid in men. About 60–70% of testosterone is bound to sex-hormone binding globulin (SHBG), while the remaining proportion is free and biologically active [42]. Free testosterone can be converted to a more potent AR activator, dihydrotestosterone (DHT) via 5*α*-reductase enzymes [43]. DHT act by stimulating osteoblast proliferation and enhancing osteoblast differentiation, which will subsequently increase bone formation rate [44]. It can also exert pro-apoptotic effects on osteoclasts that will increase the rate of osteoclast death and indirectly reduce bone resorption activity [45, 46]. Free testosterone can be converted to estrogen via aromatase enzyme found on several tissues including adipose tissue [47] and osteoblastic cells [48, 49]. 

Estradiol will then activate estrogen receptors (ERs), mainly ER*α* receptors, which are located on osteoblasts and osteoclasts. Aromatization of androgen is important as estrogen plays a significant role not only in female, but also in male skeletal homeostasis [50, 51]. Osteoporosis is associated with various inflammatory conditions such as rheumatoid arthritis, haematological diseases, and inflammatory bowel disease. Proinflammatory cytokines such as interleukin (IL)-1, IL-6, IL-7, and tumor necrosis factor (TNF)-*α* are elevated in these conditions [52]. They mediate osteoclastogenesis by stimulating osteoclast differentiation and inhibiting apoptosis of osteoclast progenitors [53, 54]. Estrogen promotes the downregulation of these proinflammatory cytokines which stimulate the apoptosis of osteoclasts [55]. Receptor activator of NF-*κβ* ligand (RANKL) plays a crucial role in osteoclasts formation. Estrogen is able to suppress RANKL production by osteoblast-lineage cells and T and B cells [56], which consequently causes the inhibition of bone resorption by osteoclasts. Other than that, estrogen also stimulates the production of osteoprotegerin (OPG), a potent antiosteoclastogenic factor. This factor blocks the binding of RANK (which is expressed on the osteoblast progenitors) to its ligand (RANKL), thus, making it a potent antagonist of osteoclastogenesis [57].

## 4. Androgen-Deficient Male Osteoporosis

Males have bigger bones and a higher amount of cortical bones than females. This might be contributed by the stimulatory effects of androgen on periosteal modelling drifts and longitudinal bone growth. In contrast, estrogen, the female predominant hormone, suppresses periosteal bone expansion and longitudinal bone growth [58, 59]. Women have a higher risk of osteoporosis due to their lower bone mass and the exposure to tremendous decline of estrogen after menopause, which will lead to aggressive bone loss. Due to their greater bone mass, men usually present with osteoporotic fractures 10 years later than women. Although, once hip fracture has occurred, men have a higher mortality and morbidity rate than women [60]. Hence, osteoporosis in men is now recognized as a significant and important public health issue [61]. The factors involved in the cause of osteoporosis in men include hypogonadism, prolonged-use of glucocorticoid, alcohol consumption, inflammatory arthritis, and family history of osteoporosis. Hypogonadism, which is a reduction in circulating androgen levels or better known as androgen deficiency, is one of the major causes of osteoporosis in most men worldwide [62, 63].

According to the Endocrine Society, approximately four million men of the worldwide population have hypogonadism but less than 200,000 received treatment [64]. Hypogonadism can be classified into primary and secondary hypogonadism. Primary hypogonadism is caused by testicular impairment due to aging and diseases such as Klinefelter's syndrome and orchitis, while secondary hypogonadism is caused by pituitary and hypothalamic dysfunction. Adult men produce 3 to 10 mg of testosterone daily that is mostly secreted by the testes. Only about 0.05 mg of testosterone and other androgens, such as dihydroepiandosterone (DHEA), DHEA-sulfate, and androstenedione, are secreted by the adrenal glands [65]. As men age, the testosterone levels decrease and SHBG levels increase. Testosterone declines gradually after the age of 40 by 0.4 to 2.6% [66]. The serum free testosterone level of an 80 year-old man is approximately 50% of that in a 20 year-old. 

The development and interpretation of animal models are essential for the study of the role of androgen hormone in male skeletal growth. The orchidectomized adult male rat has been widely used as a model for bone studies of androgen-deficient male. According to Gill et al., orchidectomy causes a fall in serum testosterone level by approximately 80% in male rats [67]. This may explain the reduction of lumbar vertebral and tibial bone volume, as well as the reduction of femoral bone mineral density, due to the acceleration of bone remodelling rate as demonstrated in previous studies [68]. Orchidectomy also results in reduced bone strength, body weight, and lean body mass [69, 70]. Recently, Nazrun et al. have shown that orchidectomy causes significant bone calcium loss which is consistent with osteoporosis due to androgen deficiency. In the same study, a bone resorption marker, C-terminal telopeptide of type 1 collagen (CTx), was found to be elevated in the orchidectomized rats [71]. This indicates that androgen plays a major role in male skeletal regulation, whereby orchidectomy may cause bone loss by increasing the bone resorption activity. 

The loss of gonadal function also causes an upregulation of osteoclastogenesis by increasing the production of IL-1, IL-6, IL-7, and TNF-*α* [72], which will result in bone resorption. As the testosterone synthesized by the gonads is the major source of circulating estradiol in males, orchidectomy will not only reduce the testosterone level, but also the estradiol levels [73]. Estrogen withdrawal following androgen deficiency will result in the upregulation of RANKL and downregulation of OPG, resulting in increased osteoclastic activity. 

Orchidectomy can also promote the upregulation of reactive oxygen species (ROS), which contribute to oxidative stress [74]. This leads to osteoblast apoptosis and promotion of osteoclast differentiation [75]. Estrogen (mainly estradiol) plays an important role as an antioxidative agent that will upregulate the glutathione reductase activity to combat the deleterious ROS activity. This results in stimulation of osteoclast apoptosis and inhibition of osteoblast apoptosis.

Based on the facts above, we can see that androgen deficiency not only impairs the function of testosterone on bone, but also indirectly impairs estrogen activities. Androgen-deficient osteoporosis has long been treated using testosterone replacement therapy (TRT), which is usually given via intramuscular injection to produce a stable testosterone level as well as improving calcium absorption to reduce bone resorption. TRT is not only painful and prone to cause infection, but its prolonged use may produce harmful side effects, such as prostate cancer, liver damage, cardiovascular diseases, and painful erections [76]. On the other hand, the oral form of testosterone is mostly deactivated by the liver and is associated with liver tumors. Transdermal testosterone in the form of cream and gel may cause transference to women and children by skin contact. This is also the most expensive form as it requires a high concentration of testosterone.

The other treatment options for osteoporosis in men are biphosphonates, which include alendronate and risedronate. These drugs have been shown to significantly reduce the risk of vertebral fracture. However, they can cause many adverse effects, such as abdominal pain, esophageal cancer, jawbone necrosis, muscle pain, and nausea [77]. The Food and Drug Administration (FDA) has also approved recombinant human parathyroid hormone (PTH) for the treatment of androgen-deficient osteoporosis. Several studies have shown that PTH has positive effects on bone turnover rate and significantly increases bone mineral density [78]. However, due to its high cost of treatment and adverse effects, such as marrow fibrosis, headache, and osteosarcoma, PTH should only be considered for men with severe osteoporosis. Another effective medication that is still under an ongoing multicenter international trial is strontium ranelate. Strontium ranelate has been proven to be significantly effective in reducing fractures especially in postmenopausal women by stimulating bone formation activity and simultaneously reducing bone resorption by osteoclasts [79]. Recent studies have found that strontium ranelate is also able to increase bone mineral density in osteoporotic men [80]. Regardless the efficacy of strontium ranelate on improving bone health, it is, however, still has not been approved by FDA and may produce side effects such as nausea, diarrhea, and thrombosis [81]. Among the mentioned available treatments, TRT is widely used in male osteoporosis due to androgen deficiency. Despite the available treatments of osteoporosis, the development of an alternative treatment agent to protect against osteoporosis in men is highly desirable. This alternative agent should ideally be without any side effects and can be easily taken as a supplement. 

## 5. Mechanism of Action of *Eurycoma longifolia *Jack in the Prevention and Treatment of Osteoporosis

The pharmacological properties of *EL* have been widely studied. Over the years, pharmacological evaluations of this plant showed that it has antimalarial [82], antibacterial [83, 84], antitumor, and antioxidant [85, 86] properties. Recently, it was established that *EL* may be used in the prevention and treatment of osteoporosis, or more specifically, male osteoporosis. In the study, it was shown that orchidectomized male rats supplemented with *EL* did not experience bone calcium loss [32]. The root of *EL* contains a wide variety of chemical compounds including alkaloids, quassinoids, quassinoid diterpenoids, eurycoma no side, eurycolactone, eurycomalactone, phenolic component, and tannins [87]. Apart from these compounds, a bioactive peptide of 4.3 kDa with aphrodisiac properties has been identified [88]. These phytochemicals may be the reasons behind the effectiveness of *EL* on various diseases. The underlying mechanism of *EL* against osteoporosis is still unclear, but studies have demonstrated that it is mainly due to its aphrodisiac property. With regard to the said aphrodisiac property, several animal model studies using rats revealed an increase of sexual motivation and performance along with the increase of serum testosterone concentrations in treated rats [89]. While animal studies do not always guarantee similar results in human, it has been shown that *EL* supplementation has increased the level of serum testosterone in most subjects in a human study [86].

The bioactive complex polypeptides from the *EL* root extract, labelled as eurypeptides, can exert and enhance their effects on the biosynthesis of various androgens [90]. Eurypeptides work by stimulating dihydroepiandosterone (DHEA). DHEA will act on androgen receptors to initiate the conversion of androstenedione and androstenediol to testosterone and estrogen, respectively [91]. These eurypeptides may also alleviate SHBG and subsequently increase the free testosterone level [92]. Due to these proandrogen properties of *EL*, it is able to stimulate osteoblast proliferation and differentiation, resulting in increased bone formation rate. High levels of testosterone and estrogen may also exert proapoptotic effects on osteoclasts, reducing the bone resorptive activity. As testosterone level decreases with age, it has been suggested that men can consume *EL* (at suitable dosage) as a supplement, to replace the famous proandrogenic drug, sildenafil (or better known as Viagra), which can cause harmful adverse effects [93]. 

Other than its proandrogenic properties, *EL* contains high level of nitric oxide (NO) [94] that have effects on bone. NO derived from the endothelial isoform of nitric oxide synthase (eNOS) is widely expressed in bone on a constitutive basis. It acts as a mediator, together with prostaglandin, to promote bone formation and suppress bone resorption [95]. NO in bone cells is stimulated by IL-1 and TNF. At low concentration, NO exerts a destructive effect to potentiate IL-1-induced bone resorption [96]. This effect appears to be biphasic as at high concentrations, NO has been shown to inhibit IL-1- and TNF-*α*-induced bone resorption [97, 98]. NO is only needed in low concentration to reduce bone resorption and enhance bone formation activity, that is why its short-acting property still makes an impact on bone. Apart from that, NO activity can be upregulated by estradiol [99]. As consumption of *EL* may increase testosterone level which later will be converted to estradiol, this will indirectly enhance the effect of NO on bone. Van't Hof and Ralston confirmed that NO is a potent inhibitor of bone resorption via two main mechanisms: by inducing osteoclast apoptosis and by inhibiting mature osteoclast activity [100]. By taking into account the NO effects on bone, the high level of NO in *EL* can be postulated to be effective in reducing bone resorption activity. 

Male osteoporosis can also be explained in terms of oxidative stress mechanism. Free radicals, mainly reactive oxygen species (ROS), are efficiently scavenged in the body. However, oxidative stress will occur when there is an imbalance between increased ROS and inadequate antioxidant activity [101]. Orchidectomy, as shown in the model of androgen-deficient osteoporosis, can promote upregulation of ROS which leads to oxidative stress. Oxidative stress plays a role in osteoblast apoptosis and osteoclast differentiation [102]. Antioxidants such as tocotrienol are known to offer protection against oxidative damage. Tocotrienol can prevent lipid peroxidation by enhancing its glutathione peroxidase (GPx) enzyme activity. Due to its antioxidative property, tocotrienol can protect bone cells from damages caused by lipid peroxidation to maintain bone remodelling [103]. It has been shown that palm vitamin E prevents bone mineral density loss due to orchidectomy [104]. According to Tambi and Kamarul, *EL* contains high concentrations of superoxide dismutase (SOD), another antioxidant that plays an important role in counteracting oxidative stress [105]. Other components of *EL*, such as alkaloids and triterpenes, can also act as antioxidants that may reduce bone loss and maintain the bone formation rate. 

## 6. Safety and Toxicity of *EL*

Although *EL* has been used in traditional medicine for generations in Malaysia, it was only in the late 1990s that researchers started to pay more attention on its safe dosage and toxicity profile. An acute toxicity study done by Satayavivad et al. has found that the oral Lethal Dose 50 (LD_50_) of the alcoholic extract of *EL* in mice is between 1500–2000 mg/kg, while the oral LD_50_ of the aqueous extract form is more than 3000 mg/kg [106]. Toxicity of *EL* was further tested by Shuid et al. in their acute toxicity study, where a dose of 5000 mg/kg of *EL* extract was given orally to rats within 24 hours. It was found that the LD_50_ for aqueous extract of *EL* was more than 5000 mg/kg [107]. To extrapolate this animal dosage to human, normalization of body surface area (BSA) method should be used, as shown below [108].

**Formula for Dose Translation Based on BSA**

Formula for dose translation from animal to human using BSA method (Source: [108])

(1) HED  (mg/kg)=Animal  dose  (mg/kg)×Animal  KmHuman  Km.    

Hence, *EL* intake is unlikely to cause fatality in human as the equivalent LD_50_ for *EL* is extrapolated to be 810 mg/kg in an adult man. In another subacute toxicity study, *EL* extract at the doses of 600, 1200, and 2400 mg/kg were given to rats, respectively, for 28 days. Pathological changes in the liver were seen in two rats, each from the group treated with 1200 and 2400 mg/kg. Clinical study in human done by Tambi has found that a dose up to 600 mg/kg did not cause any adverse effects [109]. *EL* is considered safe as long as it is not taken in high dose. Based on the results of previous toxicity studies, *EL* is normally recommended to be administered to men at the dose of 200–400 mg daily and should be used with caution, especially in the elderly. Currently, *EL* is commercially sold worldwide following this established dosage in the form of tablets for easier daily consumption.

## 7. Conclusion

Based on established literatures on health benefits of *EL*, it is important to conserve this valuable medicinal plant for the benefit of future generation. There are several mechanisms proposed for its antiosteoporotic effects. The main mechanism is via its testosterone enhancing effects for the prevention and treatment of androgen-deficient osteoporosis. Other mechanisms involved are through its nitric oxide contents and antioxidative properties. Due to its safety profile and potential as an alternative antiosteoporotic agent, further studies are warranted to document a better and conclusive mechanism for its therapeutic action.
